# About a subcutaneous calcification

**DOI:** 10.11604/pamj.2018.29.9.14188

**Published:** 2018-01-04

**Authors:** Dhia Kaffel, Hela Kchir

**Affiliations:** 1 Rheumatology Department, Kassab Institute, Manouba, Tunisia; 2 Gastroenterology Department, Rabta Hospital, Tunisia

**Keywords:** CREST syndrome, sub-cutaneous calcification, systemic sclerosis

## Image in medicine

We report a case of 62-year-old Tunisian woman with a 10-year history of a CREST syndrome (systemic sclerosis meeting the criteria of the CREST syndrome of the 1980 American College of Rheumatology classification for raynaud phenomenon, esophageal dysmotility, sclerodactyly and telangiectasia). Her daughter is treated in neurology for myasthenia gravis. Our patient presents a 5-month history of increasing inflammatory right thumb pain concomitant with the discovery of a subcutaneous hard mass (A). Radiographs of her thumb showed a sub-cutaneous calcification (as another part of the CREST syndrome criteria) (B). X-rays of hands found an acro-osteolysis (C). In the CREST syndrome, the calcific deposits can be subclinical. But, when symptomatic, it becomes painful, tender and an inflammatory reaction can occur facing the calcinosis.

**Figure 1:**
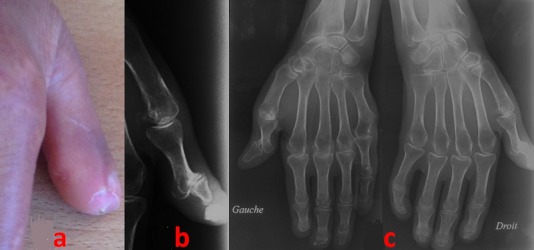
A) thickening and tightening of the skin over the thumb with deposition of calcific nodules; (B) radiographs of her thumb showing a sub-cutaneous calcification; (C) acro-osteolysis in the hands X-rays

